# Can the income level of rural residents be improved by the Chinese “Broadband Village?”: Evidence from a regression discontinuity design of the six pilot provinces

**DOI:** 10.1371/journal.pone.0248079

**Published:** 2021-04-20

**Authors:** Yang Liu, Tao Shen, Yukari Nagai, Weilong Wu

**Affiliations:** 1 School of Statistics, Southwestern University of Finance and Economics, Chengdu, China; 2 College of Design and Innovation, Tongji University, Shanghai, China; 3 Knowledge Science, Japan Advanced Institute of Science and Technology, Nomi City, Japan; 4 The Graduate Institute of Design Science, Tatung University, Taipei, Taiwan, China; Institute for Advanced Sustainability Studies, GERMANY

## Abstract

The “Broadband Village” (B&V) initiative is a substantial investment in internet construction in rural areas in six western provinces implemented by the Chinese government since 2014. This study evaluates the effect this policy has had. Panel data of 1,049 counties in China from 2015 to 2019 are used for the regression discontinuity design (RD) to estimate the impact of B&V on the improvement of the income level of rural residents. The results show that, compared to the counties without the B&V policy, the income of rural residents in counties with B&V has increased by 1.468–1.518 times, which is nearly 1.3 times the sample mean of survey data, indicating that the income level of rural residents has been improved significantly by B&V. However, the quantile regression results show that the higher the income level of rural residents, the smaller the effect of this policy. From the dynamic effect of years, the influence curve of B&V on rural residents’ income is an inverted U-shaped, first increasing and then decreasing, and the impact of this policy on the income level of highly-educated farmers is greater. Finally, three different methods are used to verify the robustness of the model.

## 1. Introduction

Many studies have proven that infrastructure contribution plays an important role in economic growth. The theoretical and practical research on income, information, and communications technology (ICT) represented by the internet is one of the methods expected to increase income. Such technology would make it easier for the poor to access information on education, health care, policy, and financial services and would help rural residents and crafters access a wider market [1–5]. The rapid development of the internet cannot be achieved without massive investment in broadband, however. In August 2013, the B&V initiative was announced by the Chinese government, establishing broadband construction as a national strategy. In 2014, the central government officially launched B&V with a 20.8 billion dollar investment that is expected to reach 98% of Chinese villages by 2020. However, few scholars have investigated the actual effect of broadband construction on Chinese rural areas. Broadband, an important infrastructure construction project in China in recent years, has an increasing coverage rate in rural areas. Therefore, the problem to be addressed in this paper is whether the income of rural residents has been increased by large-scale broadband construction.

With the policy impact of B&V implemented by the Chinese government in the western part of the country, panel data of 1,049 counties in China from 2015 to 2019 are used for the RD to estimate the impact of B&V on the increase in the income level of rural residents. Then, various heterogeneity analyses and robust tests were conducted.

This paper explores whether the income level of rural residents has been effectively improved by the R&V initiative implemented by the Chinese government in the western region since 2014. Compared to the previous literature, the marginal contribution of this paper consists of two points. The first is a recognition strategy. The RD method was used in the paper to compare the income gap between rural residents on the two sides of the B&V boundary, and the humanistic economic characteristics in counties around the boundary are almost the same so that the problem of missing variables can be better solved. Second, the research on the effect of network infrastructure on income and the influence of location-oriented policies on resident income are enriched in favor of evaluating the effect of B&V with multiple dimensions and perspectives. Evidence at the county level with a larger sample size was provided in this paper, so its conclusions are also more persuasive. Besides, the regional, educational, and year heterogeneity of the effects of B&V on the income level of rural residents was investigated.

### 1.1 Policy background

Since China provided internet access to the public in 1994, internet construction has experienced great progress in terms of the user penetration rate, internet tariff level, and average downward speed, which is largely the result of the “Broadband China” strategy actively promoted by the government. In August 2013, the “Broadband China” Strategy and Implementation Plan was formally issued by the State Council, identifying the broadband network as a strategic public infrastructure. It was also presented that broadband would be developed in three phases: the overall acceleration stage (until the end of 2013), the popularization stage (2014–2015), and the optimization stage (2016–2020), with specific goals established for every stage [6]. Because of scattered residences and relatively low household consumption ability, the cost of broadband construction in rural areas is huge, but the return is low. Therefore, the construction of rural broadband has been behind that of urban broadband for a long time [7–9]. In June 2014, with the overall planning of the goals of the “Broadband China” strategy, the B&V pilot project was conducted jointly by the National Development and Reform Commission, the Ministry of Finance, and the Ministry of Industry and Information Technology to achieve the corresponding rural broadband development goals. Based on the project plan, more than 95 percent of villages in Inner Mongolia, Sichuan, Guizhou, Yunnan, Shanxi, and Gansu provinces will be connected by optical cables by 2020, and 30 percent of rural households will have broadband access with speeds up to 4 Mbps. The pilot project of the B&V has the following characteristics. First, not only rural cable connections but also improvement of the broadband penetration rate is emphasized. Second, the upgrade of the data transmission speed, to improve the network speed, is addressed. Third, wireless broadband and wired broadband are developed at the same time, and the development of 3G, 4G, and LTE networks is accelerated to help rural residents access broadband at lower prices and faster speeds.

### 1.2 Literature review

The relationship between economic growth, social infrastructure, and information infrastructure was investigated by Northrop and Kraemer [10]. After studying factors affecting internet penetration in different countries by path analysis and multiple regression analysis, they believed that information infrastructure plays an important role in the internet penetration rate. GDP and social infrastructure also have a significant positive influence on information infrastructure. A survey of 19 countries by the OECD also shows that broadband technology penetration is closely related to economic growth [11]. With research on New Zealand from 1987 to 2001, Oxley and Carlaw believed that the profit brought by the ICT service sector in New Zealand has a significant positive relationship with GDP growth, so increasing ICT investment is of great significance for economic growth [11].

Krueger first focused on the return rate of computers and believed that the return rate of wages can be increased by 25% ~ 30% by using computers [12]. However, Dinardo and Pischke argued that the problem of missing variables in the model was not considered by Krueger’s research, causing an excessive return rate of computers [13]. In subsequent studies, the researchers analyzed the impact of computer use on income by controlling individual characteristics. Babilonia and Wulff believed that the use of computers could bring about a 10% income return [14]. Meijers built a structural model with the data of 162 countries from 1990 to 2008 and found that every 10% increase in internet use resulted in an increase of 3.9 percent in the proportion of total imports and exports to GDP, thus driving per capita GDP growth by 0.17 percent [15].

With the development of Internet and other information technology, based on the above research on the impact of Internet use on GDP, people begin to pay attention to the impact of Internet use on personal income. [14, 16] using the U.S. census data, we found that the income of Internet users at work is higher than that of non internet users, just like the impact of computer use, but different from computer use, the wage premium of Internet users has a downward trend over time. [17] also found that the income of individuals who use the Internet is higher, and this wage premium is not limited to using the Internet at work. The income of individuals who use the Internet at home is still higher than that of individuals who have not used the Internet. They believe that the productivity improvement brought by the accumulation of human capital related to information technology is the direct reason for individuals to obtain higher income.

With the rapid development of global economy and society, new network information communication technologies such as Internet and mobile phone have been widely used. [18, 19] the study found that information communication technology only has a favorable impact on those rich people with higher income and higher education level, and will gradually widen the income gap between high-income groups and low-income groups. According to the survey data of developing countries, some scholars found that the popularity of Internet and mobile phones has a significant role in improving the sales price and sales volume of agricultural products, increasing farmers’ income and improving farmers’ welfare [20]. However, some scholars have come to the conclusion contrary to the previous research through investigation and Research [21, 22] and found that information communication technologies such as Internet and mobile phone may not have a significant positive impact on farmers in developing countries. To sum up, the impact of Internet use on Farmers’ income is uncertain.

With the rapid popularization and development of the Internet and mobile Internet information technology in rural areas, an increasing number of scholars have paid attention to the impact of Internet use on the income of rural residents based on research on the impact of computer use on urban residents’ income. The study by Bank on developing countries, such as India and Burkina Faso, shows that the use of broadband Internet has a positive impact on rural income [23]. Research by Gao shows that the popularization of computers in rural areas will increase the income of rural residents over time, but its average effect is limited [24]. Xia investigated the impact of Internet diffusion on rural development [25] and Whitacre studied the influence of rural broadband diffusion on local economic growth [26].

This paper explores whether the income level of rural residents has been effectively improved by the R&V initiative implemented by the Chinese government in the western region since 2014. Evidence at the county level with a larger sample size was provided in this paper, so its conclusions are also more persuasive. Besides, the regional, educational, and year heterogeneity of the effects of B&V on the income level of rural residents was investigated.

### 1.3 Hypotheses

In theory, workers’ income is affected by the Internet in various ways. First, information resources are made available, greatly reducing information costs and eliminating information asymmetry, which improves work efficiency and income [27]. Second, the development of new industries is driven by the Internet, and more jobs are created. Many scholars argue that Internet use can increase the off-farm employment rate of farmers and further increase their income level [28–30]. Third, the problems of distance and time can be reduced by Internet information technology, facilitating communication between people. Therefore, social resources and social networks of workers accumulate, and the employment and self-employment opportunities and labor income level of workers are further improved [31–33].

Property income, wage income, and operating income of farmers are all improved by B&V through three paths: rural area, agriculture, and farmer. Specifically, the wider and deeper application of financial platforms, e-commerce platforms, and websites of government departments are promoted by the development of the Internet in rural areas. For example, rural residents can learn about and participate in financial activities, such as financial management, insurance, crowdfunding, and financing by online financial platforms, which increases their property income to some extent. The entry of “Taobao”, “Jingdong” and other e-commerce platforms changes the sale mode of agricultural products, increases sales channels, and promotes the growth of sales (operational income). Many e-commerce businessmen are indirectly trained by wide participation in e-commerce activities, increasing their wage income. Besides, the brand effect of agricultural products can be established by network sales to greatly increase the price and sales income of agricultural products. Meanwhile, Internet office government departments can improve comprehensive services in rural areas and reduce the business expenditure of farmers, the equivalent of increasing their operational income.

In the agricultural path, many aspects of agricultural production have been improved by the rapid development of emerging Internet technologies, such as the Internet of Things, big data, and cloud computing. Not only is the cost of humans and materials reduced, but production efficiency is also increased, accelerating the growth of farmers’ operational income. Besides, thanks to the rapid development of tourism website platforms such as "Ctrip" and "Tuniu", a large number of tourists are attracted to the countryside, which increases the income related to agricultural tourism.

In the path of farmers, the development of the Internet facilitates changes in farmers’ ways of thinking, improves their entrepreneurial awareness, and enriches their entrepreneurial methods. The long-term source of agricultural products established by farmers on network platforms broadens their entrepreneurial scope and types and increases their entrepreneurial income. Besides, the rise of short video apps provides farmers with the professional skills needed to show their talents to Internet users and earn profits from the fan economy. Besides, the Internet can also improve the ability of farmers, providing them with the possibility of earning more operational income. Therefore, it is assumed in this paper that B&V has a significant positive impact on rural residents’ income.

The existing literature has focused on the impact of computer and Internet use on the income of urban residents, but few studies have focused on the income of rural residents. Because the resources and geographic locations of the western region are different from those of the eastern and central regions, the treatment group and the control group are quite heterogeneous, so the estimation framework of the double-difference method is not applicable. Therefore, the RD method used in this paper is based on county-level panel data. The shortest distance from the county to the boundary of the B&V is taken as the driving variable in the RD function. Therefore, the unobservable related factors around the boundary can be captured and the endogeneity problem can be solved more effectively by clearly identifying the causal relationship between the B&V and the income level of rural residents. Finally, through a nonparametric estimation method, removing designated poverty alleviation counties and placebo testing was used to verify the stability of the model. The results show that the income level of rural residents is significantly improved by B&V, although there is heterogeneity among samples of different income levels and educational levels.

The rest of the paper is organized as follows. The second section presents the method, including the theoretical model and data explanation. The third section provides the empirical results and the heterogeneity analysis, and the main results are given in this part. The robustness test is presented in the fourth section, and the paper concludes in the final and fifth sections.

## 2. Method

### 2.1 Theoretical model

To analyze the impact of the B&V policy implemented by the Chinese government since 2014 on the income level of rural residents, a theoretical framework common in the literature [34–36] is used in this paper. Combined with the RD method, the econometric model is as follows:

$$\begin{array}{l} Y_{it}=\alpha +\beta B\&V_{i}+F\left({\mathrm{Distance}}_{i}\right)+B\&V_{i}\cdot F\left({\mathrm{Distance}}_{i}\right)+X_{it}^{\prime }\delta \\ \;+u_{p}+\lambda_{t}+\varepsilon_{it} \end{array}$$ (1)

where *i*, *p* and *t* are county-level administrative regions, provinces, and years, respectively, and the explained variable *Y*_*it*_ is the income level of rural residents in the county. The core explanatory variable of the B&V model is a dummy variable for whether the B&V policy is implemented in the county. The treatment group, the county on the left of the boundary, is 1, and the control group, on the right of the boundary, is 0. Because the Chinese B&V policy is mainly implemented in the western mountain areas, six Chinese western provinces with B&V are taken as the research object. The boundary is defined as the administrative boundary between these provinces and their neighboring provinces, referring to the literature [24].

β, the estimated coefficient of B&V, is the key to this research, which reflects the net effect of the B&V policy on the income level of rural residents. If *β*<0 and it is remarkable, the improvement of rural residents’ income is significantly prevented by the B&R. If *β*>0 and it is remarkable, the income level of rural residents is significantly improved. It *β* is not remarkable, the implementation of the B&V has little effect on the income level of rural residents.

In formula (1), Distance is a forcing variable representing the shortest distance from county i to the boundary, where the distance from the counties in the treatment group is positive, the distance from the counties in the control group is negative, and the dividing line, the breakpoint, consists of points at distance 0. *F*(Distance_*i*_) is a smooth function of Distance, which is generally defined as a low-order polynomial model on both sides of the breakpoint. B&V_*i*_⋅*F*(Distance_*i*_) is a cross term between B&V and *F*(Distance_*i*_) to make the model setting more general, which means that the low-order polynomial models on two sides of the breakpoint have different slopes. Besides, $X_{it}^{\prime }$ are control variables to control the impact of other factors on the income level of rural residents. *u*_*p*_ is the province fixed effect to control factors not changing with time among various provinces, such as the differences in geographical factors and resource endowments. *λ*_*t*_ is the year fixed effect. *ε*_*it*_ is the random error term.

There are two methods for RD estimation. Eq 1 is the RD average treatment effect estimation method, which has the problem of uncertain *F*(Distance_*i*_) function settings. Another is the local linear regression of the nonparametric estimation method, also known as the minimization function:

$$\begin{array}{l} \min_{\alpha ,\beta ,\delta ,\varphi ,\delta }\sum_{i=1}^{n}K\left[{\mathrm{Distance}}_{i}/h\right]•\\ {\left[Y_{it}-\alpha -\beta {B\&V}_{i}-\delta {\mathrm{Distance}}_{i}-\varphi {B\&V}_{i}\cdot {\mathrm{Distance}}_{i}-X_{it}^{\prime }\delta \right]}^{2} \end{array}$$ (2)

In formula (2), *K*(•) is the kernel function and *h* is the bandwidth. Referring to [37], the triangular kernel is more suitable for boundary estimation, so it is the main estimation result of local linear regression in this paper. Eqs (1) and (2), two estimation methods of RD, have their advantages and disadvantages. Some studies [37, 38] believe that local linear regression can solve the problem of slow convergence on the boundary and reduce the error of the estimated value. Others [39] found that the parametric estimation method has a problem of sensitive polynomial order selection. Therefore, local linear regression is used as the main estimation method in empirical research, supplemented by global polynomial regression of the parametric estimation method. This method was also used by various researchers [40, 41].

### 2.2 Data and variable construction

The B&V policy was launched by the Chinese government, positioning broadband as a strategic public infrastructure and coordinating wireline and wireless technology. It accelerates the introduction of broadband into Chinese rural areas and increases the popularity of Internet applications among farmers and rural areas. This policy is mainly implemented in six provinces, including the Inner Mongolia Autonomous Region, Sichuan Province, Guizhou Province, Yunnan Province, Shanxi Province, and Gansu Province. Approximately 208 billion dollars were invested in these pilot provinces by the central government, local government, and telecom enterprises until the end of 2019, and 98 percent of the villages were covered by broadband. Especially with the help of e-commerce platforms, many profitable rural e-commerce platforms spring up from agricultural e-commerce—e-commerce relating to rural areas, farmers, and agriculture. Internet infrastructure construction plays a significant role in promoting farmers’ entrepreneurial income [24, 30, 42]. The data are collected from the above six western provinces and their neighboring provinces. The sample in this paper is the panel data of 1,049 counties, county-level cities, and districts in China between 2014 and 2019. The data are provided by the "China Health and Health Commission’s Migrant Population Development Research Database", the "China County and City Social and Economic Statistical Yearbook", the "China Regional Economic Statistics Yearbook", the "China Statistical Yearbook", etc. Besides, to eliminate the influence of extreme values, the highest and lowest 1% of samples of the explained variables and control variables are removed.

1. Explained variable: Income level of rural residents. Per capita net income (RMB) is generally used by the National Bureau of Statistics to evaluate the income level of rural residents, which is also used for studies at the provincial level [43] and urban level [36] in some relevant literature. However, the per capita net income of the farmers in some county-level administrative regions is not available from the National Statistical Yearbook, so the data in this article are from the database of floating population development research in the China Health and Health Commission. The survey is conducted yearly by the China Health and Health Commission, covering 31 provinces (autonomous regions and municipalities) and the Xinjiang Production and Construction Corps. The annual population report data of all employees in the abovementioned regions are taken as the basic sampling frame, and a stratified, multistage, and scale-proportional PPS(PPS sampling is a kind of probability sampling. It means that in multi-stage sampling, especially in two-stage sampling, the probability of primary sampling units being selected depends on the size of their primary sampling units. The larger the primary sampling units are, the greater the chances of being drawn. The smaller the primary sampling units are, the smaller the probability of being drawn. It is to divide the population into units with different capacities and the same marks according to an accurate standard, and carry out the sampling with different ratios in the population.) a method is used for sampling. The subjects of the survey are residents aged 15–59 who have lived there for more than one month and are not registered in the district (county, city). The survey involved basic information on the interviewees and their employment status, social insurance, living conditions, personal income, etc. There are various reasons for adopting this database: First, the scope of this survey is broad and involves many indicators, including data on the population structure of urban and rural households, occupational background, and education level. Second, from the micro survey data, it is the survey with the longest period organized by the Chinese government. Third, there is a large difference in the survey objects. This survey has more samples, and the surveyed families are quite different in terms of geographical location, socioeconomic levels, living habits, etc., which makes the sample representative.
2. Core explanatory variable: B&V policy. The B&V variable is represented by a dummy variable in this paper. The county with a B&V policy is 1; otherwise, it is 0. Since 2014, the implementation scope of the B&V has not changed, which means that the core explanatory variable in this paper is constant. Therefore, in the control of the individual fixed effects in Eq (1), the province fixed effect is controlled.
3. Driving variables: the distance from the county-level administrative district to the boundary. Considering the RD model setting, the distance of the counties (left side of the boundary) in the treatment group is positive, and the distance of the counties (right side of the boundary) in the control group is negative. Two methods are used to calculate the shortest distance in this paper. One is to measure the shortest distance from the centroid of the area to the boundary, and the other is from the county government (administrative center) to the boundary. Since the first method is commonly used in previous studies [40, 43], it is used here for the empirical study, and the second method is used for the robustness test.
4. Control variables. To control the impact of other variables on the income gap between minorities and Han people, referring to the literature [24, 25, 39], the following control variables are introduced: sex, age, age squared, education level, state of health, marital status and engagement in off-farm activities. The definition and descriptive statistical analysis of the variables are shown in Table 1. In all samples, only approximately 59 percent of the farmers in the B&V implementation area and their average per capita income are higher than those of the farmers in the area without B&V. Compared with the existing literature using county-level panel data, there is no abnormality in the distribution of variables, which are all reasonable, ensuring that the research data are reliable.

**Table 1:** Definition and descriptive statistical analysis of variables.

Variable name	Variable symbol	Definition	Full sample		Treatment group sample		Control group sample	
			mean	SD	mean	SD	mean	SD
The income level of rural residents	*Y*_*it*_	As mentioned above	5.417	0.920	6.133	0.740	4.842	0.910
The “Broadband Village”	B&V	As mentioned above	0.570	0.022	1	—	0	—
Sex	*Gender*	Sex:	0.630	0.470	0.643	0.467	0.611	0.476
		Men = 1;						
		Women = 0						
Age	*Age*	Physical age	41.125	11.575	35.455	10.206	49.271	8.032
Age squared	Age^2^	Physical age square	1816	943	1352	761	2483	768
Education level	*Education level*	Highest level of education:	2.957	0.901	3.276	0.792	2.474	0.840
		No education = 1;						
		Primary education = 2; Secondary education = 3;						
		Senior high school education = 4;						
		College education or above = 5;						
State of health	*State of health*	The current state of health: Very unhealthy = 1;	4.005	0.922	40171	0.788	3.766	1.043
		Less healthy = 2;						
		General = 3;						
		Healthy = 4;						
		Very healthy = 5						
Marital status	*Marital status*	Current marital status: widowed = 1;	0.879	0.305	0.809	0.375	0.979	0.094
		Single = 0						
Engaged in off-farm activities or not	*Nonagricultural labor*	Whether to obtain income from off-farm labor in agricultural activities:	0.229	0.416	0.234	0.420	0.221	0.412
		Yes = 1; No = 0						

## 3. Results and discussion

### 3.1 Graph results

The precondition of using progressive RD for policy evaluation is to meet the parallel trend hypothesis; that is, before being impacted by the pilot policy, the income level in the experimental group has the same trend as that in the control group. In addition, because the B&V is affected by policy implementation intensity, policy implementation basis, and production factor adjustment, there may be a digestion period, resulting in a hysteresis quality regarding the effect of policy implementation. Therefore, the difference in income level between in the experimental group and the control group after the implementation of B&V is analyzed.

**Fig 1:**
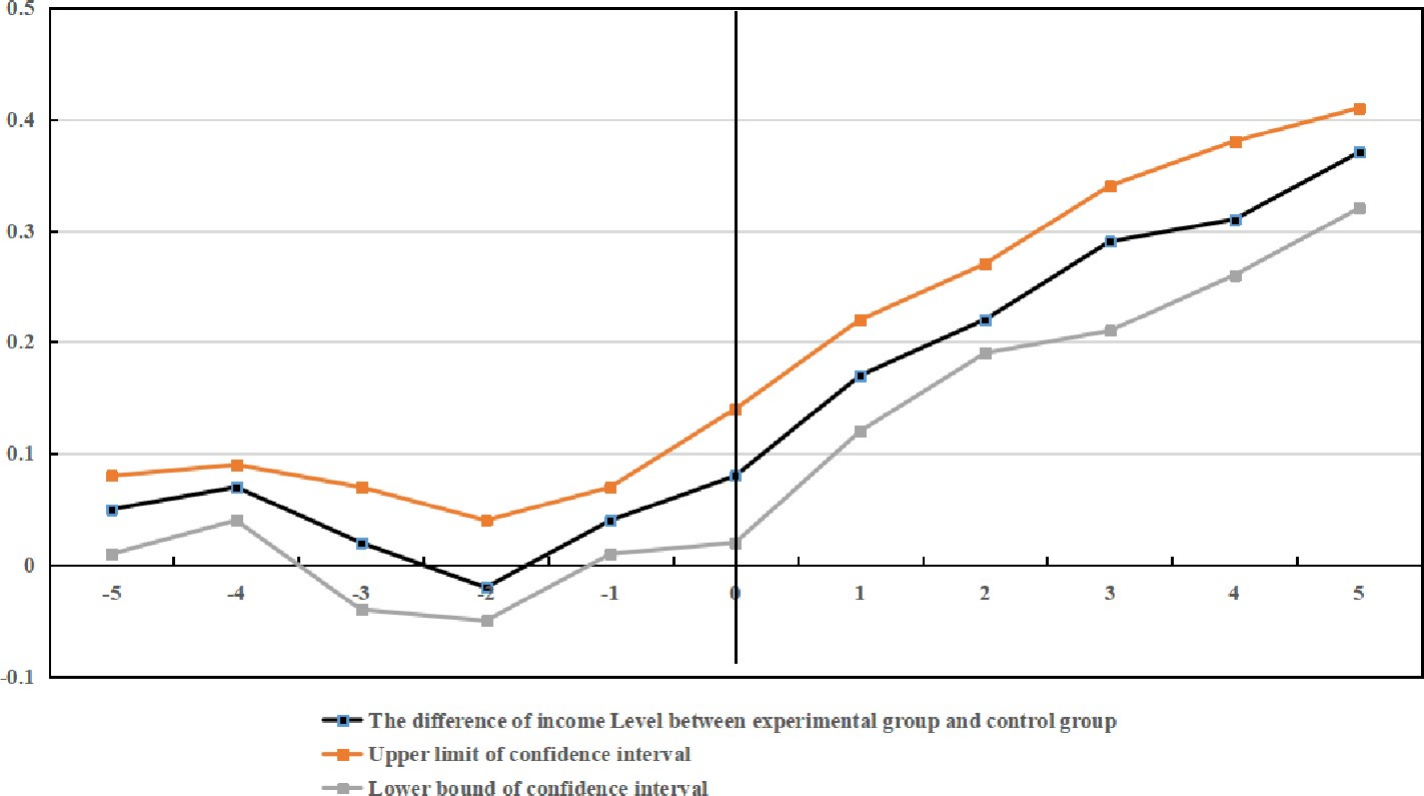
Bounce diagram of the income level of rural residents on two sides of the administrative boundary.

**Fig 2:**
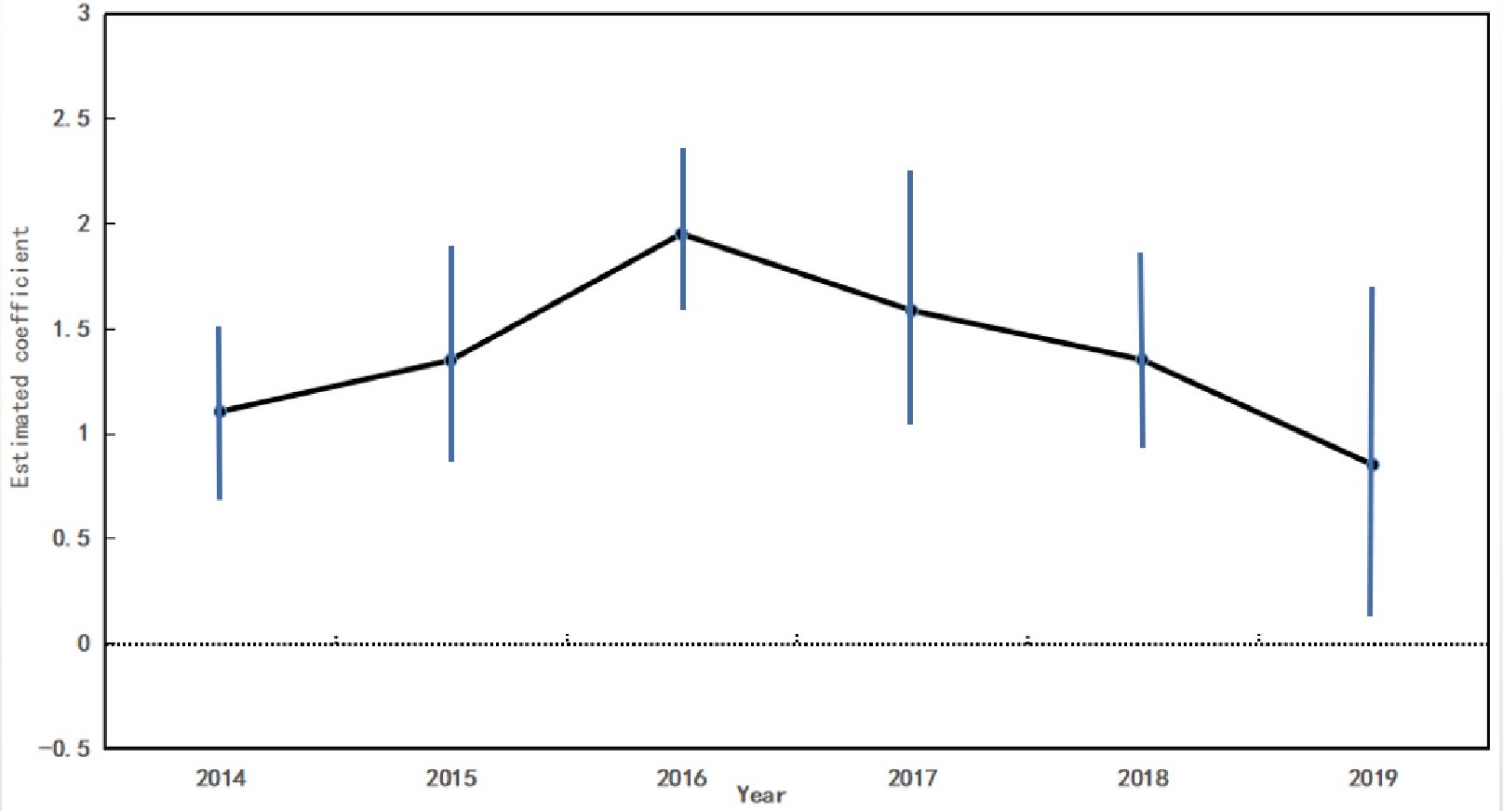
Year heterogeneity results of the effects of B&V on rural residents’ income.

The trend of income level before and after implementing B&V is compared in a graph (Fig 1). Fig 1 shows no significant difference between the experimental group and those in the control group before implementing B&V. However, their difference continues to increase after the policy is implemented, indicating that the data are sufficient to pass the parallel trend test, and the progressive RD better evaluates the influence of B&V on income level. In addition, the influence of the pilot policy on income level is not significant in the first year of policy implementation, but it rises gradually after two years. Therefore, there is certain hysteresis in its effect, but it is sustained over time. It is obvious that, at the boundary, there is a significant jump in the income level of rural residents in the county administrative areas of the treatment group, which indicates that the income level of rural residents in the counties with B&V is significantly higher than that in the areas without B&V. Fig 2 shows that the income level of rural residents has an obvious breakpoint, which preliminarily proves the causal relationship between treatment variables and outcome variables. This relationship will be discussed in detail in the empirical analysis.

### 3.2 Discussion

Table 2 shows the basic regression results of the effect of B&V on the income level of rural residents. Control variables are not added into columns (1), (3), (5), or (7), and only “income level of rural residents” is taken as the dependent variable. Control variables are added into Columns (2), (4), (6), and (8). As a reference, the estimation results of OLS are shown in columns (1) and (2). After controlling for province and time fixed effects, the estimated coefficients of B&V are all positive and significant at the 1% level, indicating that the income level of rural residents is widened dramatically by the implementation of B&V. When the control variables are added, the estimated coefficient of B&V decreases but is still significantly positive. On this basis, the estimation results of local linear regression by the RD method are shown in columns (3) to (8) of Table 2. The kernel functions are triangle kernel, Epanechnikov kernel, and homogeneous kernel in turn. The regression results show that the estimated coefficient of B&V is greater than 0 in all six models and meets the significance test of the 1% level, regardless of which kernel function is adopted or whether the control variable group is added. Therefore, the B&V policy implemented by the Chinese government is conducive to improving rural residents’ income, which means that the B&V policy has an improvement effect on the income level of rural residents. The estimated coefficient value shows that, under the same conditions, the income level of rural residents in counties with B&V has increased by 1.468–1.518 on average compared with that in county-level administrative regions in the control group.

**Table 2:** Basic regression results of the effect of B&V on the income level of rural residents.

	OLS		RD					
	(1)	(2)	(3)	(4)	(5)	(6)	(7)	(8)
B&V	2.749*** (**0.161**)	1.942*** (**0.672**)	1.967*** (**0.681**)	1.468*** (**0.871**)	1.861*** (**0.782**)	1.481*** (**0.571**)	1.917*** (**0.874**)	1.518*** (**0.764**)
*Gender*		0.481** (0.842)		0.584** (0.515)		0.482*** (0.874)		0.584*** (0.149)
*Age*		0.244*** (0.499)		0.278*** (0.875)		0.187**** (0.944)		0.167*** (0.878)
Age^2^		-0.002*** (0.882)		-0.003** (0.768)		-0.002*** (0.967)		-0.004** (0.875)
*Primary school*		0.276** (0.876)		0.267*** (0.556)		0.278*** (0.575)		0.287*** (0.694)
*Junior high school*		0.315** (0.153)		0.302*** (0.168)		0.317** (0.516)		0.325*** (0.846)
*High school*		0.384*** (0.682)		0.395** (0.842)		0.377** (0.653)		0.371** (0.648)
*Bachelor degree and above*		0.411** (0.665)		0.402** (0.994)		0.400*** (0.517)		0.401*** (0.665)
*State of health*		0.068** (0.587)		0.157*** (0.674)		0.248** (0.264)		0.125** (0.247)
*Marital status (Married = 1)*		-0.034 (0.157)		-0.157 (0.894)		-0.031 (0.268)		-0.034 (0.597)
*Nonagricultural labor (Yes = 1)*		0.337** (0.246)		0.365*** (0.334)		0.157** (0.357)		0.345** (0.578)
Province fixed effect	Fixed	Fixed	Fixed	Fixed	Fixed	Fixed	Fixed	Fixed
Time fixed effect	Fixed	Fixed	Fixed	Fixed	Fixed	Fixed	Fixed	Fixed
The amount of data	24658	24658	24658	24658	24658	24658	24658	24658
R^2^	0.64	0.61	0.58	0.48	0.51	0.49	0.47	0.42

Note: The values in brackets are robust standard errors

*, **, ***, which are significant at the 10%, 5%, and 1% significance levels, respectively.

Table 2 also shows the effects of other variables on farmers’ income. Among the individual characteristics, sex has a significant positive influence on the income of rural residents. The age coefficient is positive, but the age squared coefficient is negative, indicating that there is an inverted U-shaped linear relation between the income of rural residents and their age. The education level also has a remarkable positive influence on the income of rural residents, among which the effect on farmers with a college education or above is the largest. The possible reason is that farmers with higher education have higher human capital, which improves work efficiency and thus increases farmers’ income. The state of health also has a large positive impact on farmers’ income, indicating that physical health is conducive to improving farmers’ income. The effect of employment status on farmers’ income is significantly positive, indicating that nonagricultural labor in agricultural activities is beneficial to the increases in farmers’ income.

### 3.3 Model validity test

When RD is used for causal recognition, it must be the local randomization hypothesis and the continuity hypothesis. In the local randomization hypothesis, driving variables cannot be controlled precisely by economic individuals. Because the geographical breakpoint is adopted in this paper and every county-level administrative district in China has a clear position on the map, it is impossible to divide counties on the left side of the boundary to the left side (control group) to enjoy the B&V policy [44].

The second identification hypothesis is the continuity hypothesis, which means that all factors affecting the income of rural residents are continuous at the boundary, except for the B&V policy. If it is not satisfied, the impact of other variables on rural residents’ income is also taken into account in the estimated results of the RD model, so that the jump in rural residents’ income cannot be fully attributed to the implementation of the B&V policy, which can result in estimation errors. Referring to the methods by Meng and Lingsheng [45], the RD model was used to test the continuity of control variables in this paper. The results of local linear regression by the trigonometric kernel function are shown in Table 3. When seven control variables are taken as explained variables, the estimated coefficients are not remarkable, indicating that there is no significant difference between the control variables at the boundary and no jump, which satisfies the hypothesis conditions and indicates that the model is continuous. This may be because, although most of the boundaries of the B&V policy coincide with those of provinces, most provinces do not have large mountains or large rivers as boundaries, resulting in interlocking [46]. Similar economic and cultural features on both sides of the boundary are also explained.

**Table 3:** Model validity test results.

	B&V	Province fixed effect	Time fixed effect	R^2^
*Gender*	0.1357 (0.3157)	Fixed	Fixed	0.27
*Age*	0.0345** (0.1352)	Fixed	Fixed	0.18
Age^2^	-0.0038 (0.2574)	Fixed	Fixed	0.31
*Primary school*	0.0357 (0.2649)	Fixed	Fixed	0.49
*Junior high school*	0.0352 (0.0357)	Fixed	Fixed	0.26
*High school*	0.0349 (0.0385)	Fixed	Fixed	0.21
*Bachelor degree and above*	-0.0035(0.682)	Fixed	Fixed	0.22
*State of health*	0.0349 (0.2499)	Fixed	Fixed	0.18
*Marital status (Married = 1)*	0.0328 (0.5874)	Fixed	Fixed	0.20
*Nonagricultural labor (Yes = 1)*	-1.0472 (0.9494)	Fixed	Fixed	0.31

Note: The values in brackets are robust standard errors

*, **, ***, which are significant at the 10%, 5%, and 1% significance levels, respectively.

### 3.4 Heterogeneity analysis

#### 3.4.1 Heterogeneity of different income levels and education levels

The overall impact of B&V on the income of rural residents is analyzed as above, but the potential quantile difference may be explained by the overall analysis based on the sample; that is, the impact of B&V on the income of rural residents may be different for residents with different income levels and education levels. Therefore, the sample is divided into four categories according to the degree of difference in rural residents’ income: areas of low-income level (less than 20% quantile), areas of low- and middle-income level (between 20% ~ 50% quantile), areas of middle- and high-income level (between 50% ~ 80% quantile) and areas of high-income level (more than 80% quantile). Then, the sample is divided into three groups based on education level: primary education and below, secondary education, and college education and above.

In this paper, the results of local linear regression by the trigonometric kernel function are shown in Table 4. It can be found that the estimated coefficient of B&V in the sample of low income, middle and lower-income as well as middle and high income is positive and continuously decreases, which also passes the significance test of the 5% level. This indicates that, in the sample where rural residents’ income is lower than the 80% quantile, rural residents’ income is effectively improved by B&V, but the effect decreases with increasing income. In contrast, in the sample of high income, the estimated coefficient of B&V is remarkably negative, indicating that rural residents’ income is reduced by B&V in the sample in which the income level of rural residents is higher than the 80% quantile. In conclusion, the impact of B&V on the income of rural residents varies with the income level, which is small for the richest 20% of farmers.

**Table 4:** Quantile heterogeneity results of the effects of B&V on rural residents’ income.

	B&R	Province fixed effect	Time fixed effect	R^2^
Low-income level(Less than 20% quantile)	0.786** (0.841)	Fixed	Fixed	0.48
Low and middle income level(20%—50% quantile)	0.437** (0.944)	Fixed	Fixed	0.32
Upper middle income level(50%—80% quantile)	0.318*** (0.766)	Fixed	Fixed	0.52
High income level(More than 80% quantile)	-0.149*** (0.915)	Fixed	Fixed	0.40
Elementary school and below	0.024 (0.931)	Fixed	Fixed	0.28
Middle school	0.149**(0.894)	Fixed	Fixed	0.31
University and above	0.221**(0.984)	Fixed	Fixed	0.35

Note: The values in brackets are robust standard errors

*, **, ***, which are significant at the 10%, 5%, and 1% significance levels, respectively.

Table 4 shows that the sample of “primary education and below” fails to pass the significance level test, but the coefficients of “secondary education” and “college education and above” are positive and pass the significance test at the 5% level. It is worth noting that the improvement effect of B&V on rural residents’ income increases largely with the development of education. B&V has more influence on highly-educated farmers. The possible reason for this outcome is that the Internet is mainly used to obtain information for farmers, and highly-educated farmers are more skilled than farmers with less education, so they have better access to information. With the Internet, information costs are reduced, and the efficiency of the work or business is also improved; as a result, their income level is higher than that of low-educated farmers.

#### 3.4.2 Year heterogeneity of B&V

Although it is shown in the above basic regression results that the income level of rural residents has been widened by the implementation of B&V policy, it is the result of the average treatment effect, while the dynamic characteristics of different years are ignored. B&V is a national strategy of the Chinese government and its role in regional growth is influenced by supporting policies and the implementation experience of local governments. With the implementation of B&V, the related regulatory policies are gradually improved and the policy implementation capacity of local governments is also enhanced. It can be expected that the effect of B&V on the income level of rural residents is heterogeneous in years. To test this heterogeneity, following the approach of [41], regression was carried out for Eq (2) using the sectional data of the year. The estimated coefficient of each year and its 95% confidence interval are shown in Fig 2.

Fig 2 shows that from 2014 to 2019, the estimated coefficient of B&V is greater than 0, and the estimated value is between 0.5 and 2.0. From the perspective of the time trend, the improvement effect of B&V on rural residents’ income has an inverted U-shaped trend, first increasing and then decreasing. More precisely, the effect of B&V peaks in 2016, and then the marginal effect of the policy decreases gradually.

## 4. Robustness test

### 4.1 Nonparametric estimation method

The RD estimation method involves a nonparametric estimation method and a parametric estimation method. The local linear regression method based on Eq (2) is used in previous empirical research and is a nonparametric estimation method. To test whether the above conclusions are robust to different estimation methods, the parameter estimation method based on Eq (1) will be used in this section, and the regression results are shown in Table 5, where the model from column (1) to column (4) is a full-sample polynomial regression, and the polynomial orders are first, second, third, and fourth. In the four models, the estimated coefficient of B&V is significantly positive at the 1% level, and the value is also closer to the results of the local linear regression, which confirms the above conclusion. Furthermore, the sample in columns (5) to (7) is further restricted, and three bandwidths of 100 km, 200 km, and 500 km are set. Quadratic polynomial linear regression is used. The regression results show that the estimated coefficient of B&V is still significantly positive.

**Table 5:** Estimation results of the nonparametric estimation method.

	(1)	(2)	(3)	(4)	(5)	(6)	(7)	(8)	(9)	(10)
B&V	1.610*** (0.865)	1.435*** (0.684)	1.747*** (0.538)	1.605*** (0.563)	1.635*** (0.663)	1.207*** (0.648)	0.477*** (0.949)	1.476*** (0.587)	1.856*** (0.649)	1.765*** (0.865)
Control variables	Fixed	Fixed	Fixed	Fixed	Fixed	Fixed	Fixed	Fixed	Fixed	Fixed
Province fixed effect	Fixed	Fixed	Fixed	Fixed	Fixed	Fixed	Fixed	Fixed	Fixed	Fixed
Time fixed effect	Fixed	Fixed	Fixed	Fixed	Fixed	Fixed	Fixed	Fixed	Fixed	Fixed
Degree	First	Second	Third	Fourth	Second	Second	Second	-	-	-
Bandwidth	All	All	All	All	100 km	200 km	500 km	All	All	All
R^2^	0.42	0.48	0.37	0.49	0.52	0.52	0.49	0.49	0.36	0.48

Note: The values in brackets are robust standard errors

*, **, ***, which are significant at the 10%, 5%, and 1% significance levels, respectively.

Second, the per capita net income of residents is taken as the proxy variable of the income level to build the model based on Eq (2), and the results are listed in column (8). Similarly, the estimated coefficient of B&R is significantly positive at the 1% significance level. Third, all explanatory variables are postponed for one stage to reduce the hysteresis of policy implementation and the error of the simultaneous equation. The results are shown in Column (9). Fourth, PSM-DID is used for re-estimation in this paper, and the results are listed in column (10), which are similar to those before. Therefore, it coincides with the core conclusion that rural residents’ income is improved by B&R and proves that the above conclusions are robust to different estimation methods.

### 4.2 Removing the sample of designated poverty alleviation counties

Since December 2015, the government-dominated “Poverty Alleviation Plan" has been implemented from top to bottom by the Chinese government, and 592 designated poor counties have been identified. The plan promoted a significant increase of transfer payments by the central and provincial governments to poor counties, so poorer counties could receive financial support [22], greatly increasing per capita income [45]. Considering that most designated poor counties are in western China and overlap with county-level administrative regions with the B&V policy, the support policies of governments at all levels for designated poor counties may change the effect of the B&V policy on the income level of rural residents. Therefore, the regression was performed again after the sample of nationally designated poor counties was removed, and the results are shown in Table 6. It was found that the estimated coefficients of B&V are all greater than 0, in line with the previous core conclusion.

**Table 6:** The regression result of removing designated poor counties.

B&V	1.968*** (0.837)	1.615*** (0.647)	0.749*** (0.678)
Control variables	Fixed	Fixed	Fixed
Province fixed effect	Fixed	Fixed	Fixed
Time fixed effect	Fixed	Fixed	Fixed
R^2^	0.52	0.50	0.48

Note: The values in brackets are robust standard errors

*, **, ***, which are significant at the 10%, 5%, and 1% significance levels, respectively.

### 4.3 Placebo test

Although observable control variables are proven to meet the continuity hypothesis at the boundary in the above research, there may be some unobservable variables affecting the income level of rural residents, which cannot be directly tested. To rule out the possibility that the impact of the B&V policy on the income level of rural residents is disturbed by omitted variables, a placebo test was conducted by setting a false boundary line. Specifically, the true boundary line was first moved to the west by 300 km, and the bandwidth was set as 100 km. Therefore, the fake treatment group and the fake control group are on the west of the real boundary, and both enjoy the B&V policy. Therefore, the income level of rural residents on both sides of the “false boundary” is not observed. Columns (1) and (2) of Table 7 report the estimated results of driving variables measured by centroid and administrative center, respectively. The estimated coefficients of false B&V are not remarkable, indicating that there is no difference in the income level of rural residents on both sides of the false boundary.

**Table 7:** Placebo test results.

	Centroid	Administration center
B&V	0.025 (0.794)	-0.018 (0.647)
Control variables	Fixed	Fixed
Province fixed effect	Fixed	Fixed
Time fixed effect	Fixed	Fixed
R^2^	0.71	0.60

Note: The values in brackets are robust standard errors

*, **, ***, which are significant at the 10%, 5%, and 1% significance levels, respectively.

## 5. Mechanism analysis

The improvement of rural broadband construction on farmer income is first reflected in information provision, including entrepreneurial opportunity information, financial information, and information brought by the expanded social network. Residents may be attracted by information due to broadband construction, so rural consumption opportunities can be increased. They can also earn more income from the wholesale and retail of entrepreneurship. Second, because of broadband construction, more local agricultural products are sold on Internet platforms. Therefore, various mediating effect models were used to verify the existence of mediating effects.

First, for the first mechanism, cooperation between farmers and e-commerce, related online shopping services and offline stores are provided, and the wholesale and retail of entrepreneurship is also encouraged. Then, home business is classified as follows: The wholesale and retail industry is defined as 1, and others are defined as 0. The influence of broadband on wholesale and retail enterprises is estimated. Column (1) in Table 8 shows that the regression coefficient is 0.172, significant at the 1% level, indicating that the growth of wholesale and retail enterprises is greatly promoted by broadband construction. Finally, for the second possible mechanism, farmers directly sell local featured products with the help of broadband construction, large-scale cultivation will be facilitated, and products can be sold on the network. Therefore, if this mechanism works, the mediating effect of large-scale cultivation may be tested. Due to agricultural scale cultivation lacking unified standards, samples of total agricultural output values of 6,300 dollars (90% quantile) and 12,750 dollars (95% quantile) are used as the classification criteria, and large-scale cultivation is defined as higher than the above output value. The results are shown in Table 8. Estimated results of large-scale cultivation with more than 6,300 dollars are listed in column (2). It is shown that broadband construction promotes the growth of scale farming at the 1% level. Column (3) shows the estimated result of large-scale cultivation with more than 12,750 dollars. Thanks to broadband construction, scale farming is increased by 5.2 percentage points, which is significant at the 5% level. Therefore, the probability of large-scale cultivation is improved by broadband construction.

**Table 8:** The effect of broadband construction on wholesale retail and scale cultivation.

	China Wholesale & Retail industry	Family scale cultivation	
	(1)	(2)	(3)
Coefficient	0.172*** (0.265)	0.049*** (0.894)	0.521**(0.248)
Control variables	Fixed	Fixed	Control variables
Province fixed effect	Fixed	Fixed	Province fixed effect
Time fixed effect	Fixed	Fixed	Time fixed effect

Note: The values in brackets are robust standard errors

*, **, ***, which are significant at the 10%, 5%, and 1% significance levels, respectively.

## 6. Conclusion

The B&V policy, an important location-oriented policy in China started in 2010, has been officially implemented by the Chinese government since 2014, and large funds have been invested in the Internet infrastructure and the development of the Internet industry in rural areas in provinces with B&V. However, existing studies have paid little attention to the impact of B&V implementation on the income of rural residents, and it is difficult to identify the problem of missing variables in the identification of causal relationships between them. Therefore, B&V is taken as a quasi-natural experiment using panel data from 1,049 counties in China from 2015 to 2019, and the RD method was used to analyze the effect of B&V on the income level of rural residents. Then, heterogeneity was analyzed. Finally, a nonparametric estimation method, removing designated poverty alleviation counties and placebo testing, was used to verify the stability of the model. The results show that (1) overall, compared to the counties without the B&V policy, the rural residents’ income in counties with B&V is increased by 1.468–1.518 times, which is nearly 1.3 times the sample mean of survey data, indicating that the increase in rural residents’ income is affected significantly by B&V. (2) The improvement effect of B&V on the income of rural residents is remarkable, but it decreases with the increase in rural residents’ income and is small on the richest 20% of farmers. (3) From the perspective of the dynamic effects of the year, from 2014 to the present, the effect curve of B&V on the income level of rural residents is inverted U-shaped and first increases and then decreases. Specifically, after the effect of B&V peaks in 2016, the marginal effect gradually decreases. (4) The B&V encourages farmers to engage in wholesale and retail entrepreneurship and large-scale planting of agricultural products to sell on the Internet, which increases their income.

## Supporting information

S1 Data (XLS)
